# Research on application of tumor treating fields in glioblastoma: A bibliometric and visual analysis

**DOI:** 10.3389/fonc.2022.1055366

**Published:** 2022-11-10

**Authors:** Xue Du, Chunbao Chen, Yu Xiao, Yu Cui, Lu Yang, Xiaochun Li, Xueping Liu, Ruisi Wang, Bangxian Tan

**Affiliations:** ^1^ Department of Oncology, Affiliated Hospital of North Sichuan Medical College, Nanchong, Sichuan, China; ^2^ Department of Clinical Medicine, North Sichuan Medical College, Nanchong, Sichuan, China; ^3^ Department of Radiation Oncology, Fujian Cancer Hospital, Fujian Medical University, Fuzhou, China

**Keywords:** glioblastoma, tumour treating fields, bibliometrics, visual analysis, citespace

## Abstract

**Background:**

Glioblastoma, one of the common tumors of the central nervous system (CNS), is prone to recurrence even after standard treatment protocols. As an innovative physiotherapy method emerging in recent years, the tumor treating fields (TTFields) technique has been approved for the treatment of glioblastoma due to its non-invasive and portable features. The purpose of this study is to visualize and analyze the scientific results and research trends in TTFields therapy for glioblastoma.

**Methods:**

Publications related to TTFields therapy for glioblastoma were searched in the Web of Science Core Collection (WoSCC) database in September 2022. A bibliometric and visual analysis of publications in this field was performed mainly using CiteSpace and R software for country/region, author, journal, reference and keyword.

**Results:**

A total of 618 publications in this field were retrieved, and 248 were finally obtained according to the search criteria, including 159 articles (64.11%) and 89 reviews (37.89%). The cumulative number of publications increased year by year, with an average growth rate (AGR) of 28.50%. The test results of Pearson correlation coefficient showed a high positive correlation between publications and citations (r=0.937, p<0.001). The USA had the largest number of publications (123, 49.60%), followed by Germany (32, 12.90%) and China (30, 12.10%). As for the country/region collaborations, the USA cooperated most closely with other countries/regions, followed by Germany and China. The degree of collaboration (DC) between countries/regions was 25.81%. The institutions with the largest number of publications were Tel Aviv Univ (10), Harvard Med Sch (10) and Novocure Ltd (10). Moreover, Wong E (18) possessed the greatest number of publications, followed by Weinberg U (11) and Kirson E (10). The DC between authors was 97.58%. STUPP R (236) was the most cited author followed by KIRSON ED (164) and GILADI M (104). JOURNAL OF NEURO-ONCOLOGY (22) was the journal with the largest number of published publications (75), followed by FRONTIERS IN ONCOLOGY (15) and CANCERS (13). The top 10 keywords that occurred frequently included glioblastoma (156), tumor treating field (152), temozolomide (134), randomized phase III (48), brain (46), survivor (46), cancer (44), trial (42), alternating electric field (42) and radiotherapy (36). Furthermore, cluster analysis was performed on the basis of keyword co-occurrence, and finally 15 clusters were formed to determine the current research status and future development trend of TTFields therapy for glioblastoma.

**Conclusion:**

TTFields has been increasingly known as the fourth novel physical anti-tumor therapy in addition to surgery, radiotherapy and anti-tumor drugs. Cooperation and communication between countries/regions need to be enhanced in future research. Several studies have demonstrated the therapeutic potential of TTFields in glioma, and its application alone or in combination with other treatments has become a current research hotspot.

## Introduction

Gliomas are primary brain tumors derived from neuroglial stem or progenitor cells (1). Based on their histological appearance, gliomas are traditionally classified as astrocytic, oligodendroglial or epithelial tumors, with WHO grades I-IV indicating different degrees of malignancy (2). In particular, glioblastoma is the most aggressive and common form of brain tumor in adults, characterized by poor survival and high tumor (both inter- and intra-tumoral) heterogeneity (3). The complexity caused by molecular heterogeneity within the neoplasm can generate resistance to treatment and stimulate frequent recurrence, and the heterogeneity of the intratumoral cells is produced in fraction by glioblastoma stem cells (4). The conventional treatment of glioblastoma consists of complete resection of the lesion, radiotherapy for the tumor lesion area, and temozolomide (TMZ) chemotherapy as standard treatment (Stupp treatment) (5). glioblastoma stem-like cells (GSCs) are resistant to conventional treatment (4), resulting in unconspicuous improvement in the survival outcome of glioblastoma patients (6). In recent years, the development of immunotherapy and targeted therapies as well as the progress of various clinical trials has benefited the survival of glioblastoma patients (7). At the same time, emerging novel therapies such as tumor treating fields (TTFields) therapy have been applied to the treatment of newly diagnosed and recurrent brain gliomas.

TTFields (Optune^®^), a portable, non-invasive device that generates an electric field through an electric field patch applied to the scalp to exert an anti-tumor effect, is currently used in the clinical treatment of glioblastoma (8, 9). TTFields is designed with low intensity, intermediate frequency, and alternating electric fields to disrupt cell division and inhibit tumor growth (10). It was shown in earlier experiments that exposure of various tumor cell lines to TTFields exerted an inhibitory effect on tumor growth by inducing cell cycle arrest and apoptosis without any effect on non-dividing cells (11). The results observed *in vitro* were also confirmed through *in vivo* experiments on animal models, demonstrating the inhibitory effect of TTFields on the growth of solid tumors, as well as the possible clinical benefit of TTFields in preventing metastatic spreading of primary tumors (12). Further research has shown that the inhibitory effect of TTFields on growth is mainly due to anti-mitosis, and the application of TTFields can reduce the ratio between polymeric and total tubulins, preventing the normal assembly of mitotic spindles (13). In addition to the anti-mitotic effects, TTFields interferes with many biological processes, including delaying DNA repair, enhancing autophagy, inhibiting cell metabolism and angiogenesis, and limiting cancer cell migration (14, 15). TTFields enhances intra-tumoral anti-tumor immunity and increases the permeability of the cell membrane and blood-brain barrier (BBB), making it a fourth anti-tumor treatment following surgery, radiotherapy, and anti-tumor drugs (16, 17).

Bibliometrics is defined as the measurement of all aspects related to the publication and reading of books and documents (18). Bibliometrics applies mathematical, statistical and related measurement methods to evaluate literature in related fields in both qualitative and quantitative manners. Using literature data as a point of departure for analysis, it accurately captures the development trends and research hotspots in a field and provides a reference for scientific researchers in related fields. TTFields is gradually gaining familiarity as a new weapon in tumor treatment. However, no bibliometric research on the field of TTFields therapy for glioblastoma has been conducted to date. In this study, a bibliometric and visual analysis of online publications in this field over the last decade was performed by multiple bibliometric tools to obtain an overview of the current status and trends of research in this field.

## Materials and methods

### Data source

**Figure 1:**
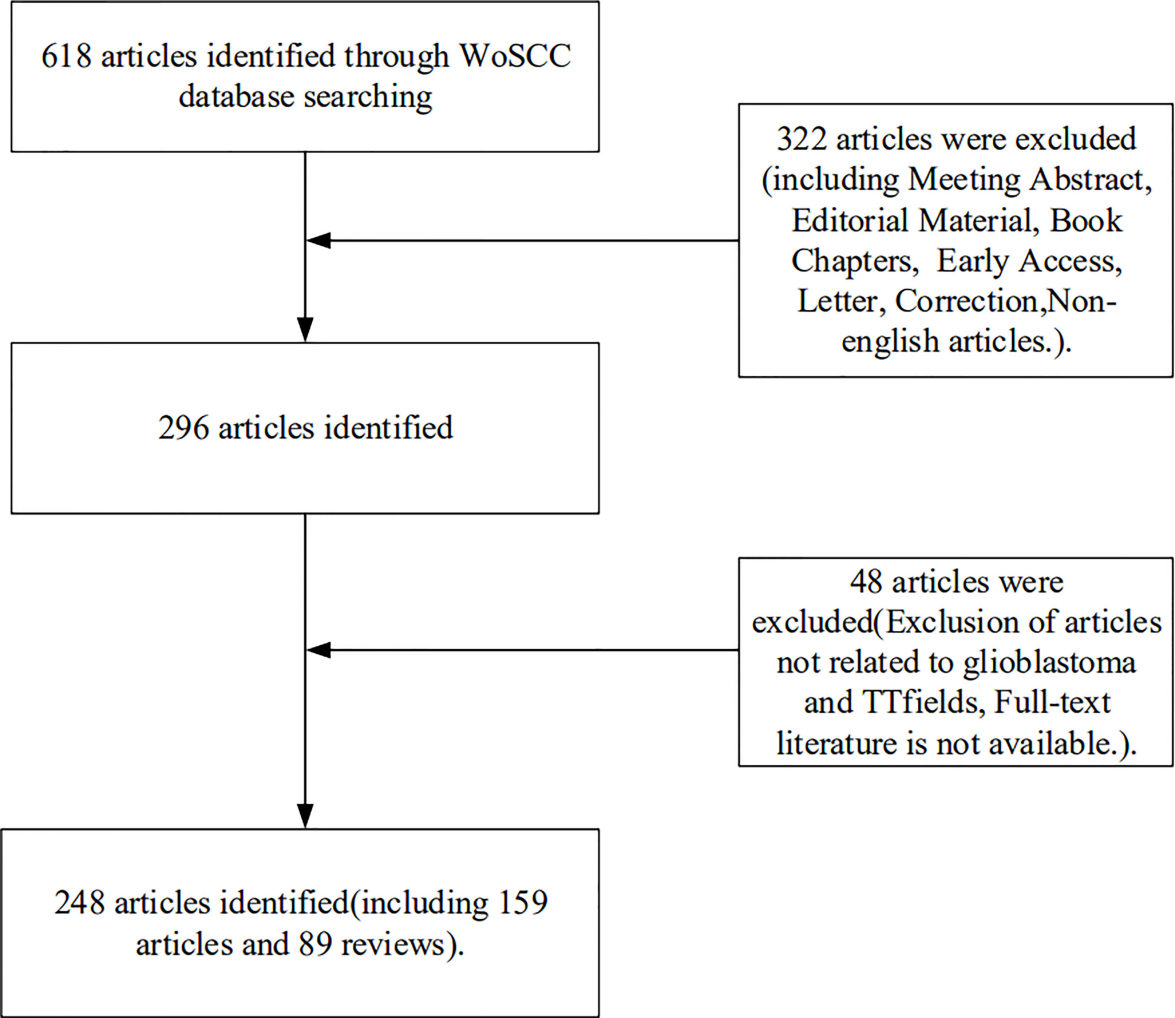
Flow chart of the literature selection process.

The Web of Science Core Collection (WoSCC) is an essential database for accessing global academic information and contains important academic journals in various subject fields. In September 2022, the literature for studies associated with TTFields therapy for glioblastoma was searched in the WoSCC database, with the search date set from 2007 to September 2022. The following search strategy was adopted: TS= (“Tumor Treating Fields”)) OR TS=(TTFields)(((((TS=(glioblastoma*)) OR TS=(“glioblastoma multiform*”)) OR TS=(“malignant glioma”)) OR TS=(“brain cancer”)) OR TS=(gliosarcoma)) OR TS=(spongioblastoma). Literature inclusion criteria (1): TTFields therapy for glioblastoma was the subject; (2) the types of literature included articles and reviews; and (3) the language of the literature was English. Exclusion criteria were as follows: (1) articles were conference abstracts, news, case studies, etc.; or (2) literature for which the full text was not available. All retrieved publications were evaluated and selected by two reviewers, and any disagreements were resolved through discussion until reaching consensus. The flow chart for selecting the literature is illustrated in **Figure 1**.

### Analysis methods

A bibliometric and visual analysis was performed for the literature in the field of TTFields therapy for glioblastoma mainly *via* CiteSpace (version 6.1.3) and R software (version 4.1.3), and Microsoft Excel 2019 was adopted for data management and trend analysis of publications. CiteSpace is a kind of bibliometric software developed by Prof. Chaomei Chen that visualizes the relationship between literature into a scientific knowledge map (19). Betweenness centrality is defined for each node in the network, which measures the shortest path through any of the nodes (20). In general, centrality ≥ 0.1 indicates that the node is more important, and nodes with high centrality are shown as purple rings in the CiteSpace visual mapping. The “bibliometrix” package in R software was used to visualize and analyze the source journals of the publications.

### Statistical analysis

Statistical processing of the data was performed using SPSS (IBM SPSS Statistics 27). p<0.05 indicated a statistically significant difference.

## Results

### Publication trends

**Figure 2:**
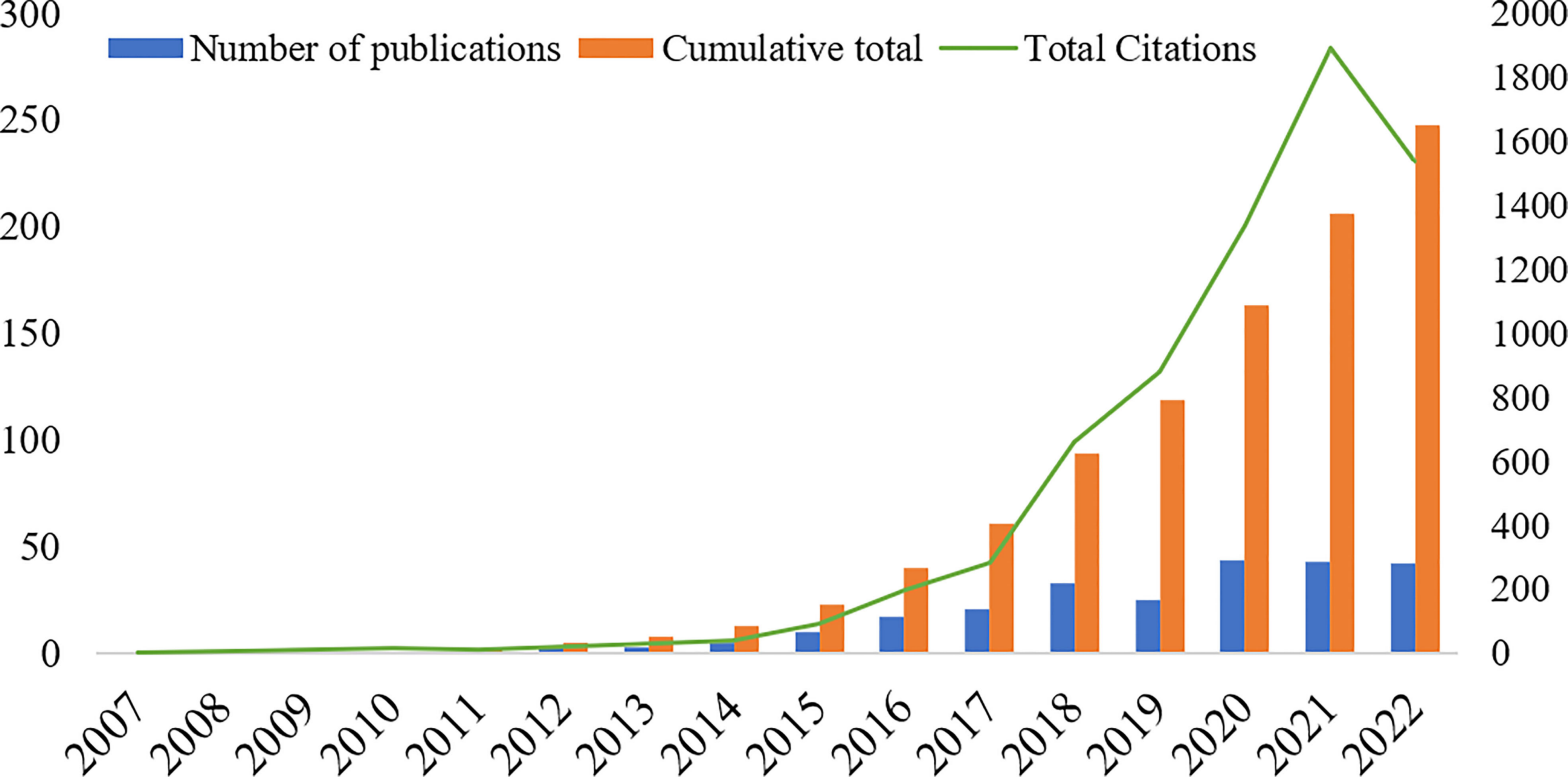
Number of annual publications, annual cumulative number of publications and annual total citations of related literature from 2007 to September 2022.

A total of 618 publications related to the field of TTFields therapy for glioblastoma published from 2007 to September 2022 were retrieved in WoSCC database, and 248 were finally obtained according to the determined search criteria, including 159 articles (64.11%) and 89 reviews (37.89%) (**Figure S1A**). **Figure 2** shows the trend of publications, including annual and cumulative publications. There were few relevant publications in 2007-2011, less than 10 publications in 2012-2014, a steady increase in 2015-2022, and more than 40 publications per year after 2020. The average growth rate (AGR) from 2007 to 2021 was 28.50%. The compound annual growth rate (CAGR) (21) of publications gradually increased from 0% in 2008 to 16.50% in 2012 and decreased to 1.57% in 2021 (**Table S1** and **Figure S1B**). Although the annual production was increasing year by year, the CAGR was basically on a decreasing trend. It was observed from **Table S2** and **Figure S1C** that the relative growth rate (RGR) increased during 2011 (69.31%) and 2012 (91.63%) and gradually decreased during 2013 and 2021, reaching 23.41% in 2021. There is a direct equivalence between the RGR and doubling time (DT) (21). The DT increased from 1.00 in 2011 to 2.96 in 2021. Further, a Pearson correlation analysis was performed, in which the correlation between publications and citations was tested by Pearson’s correlation coefficient, and a p<0.05 signified a significant correlation. The results showed a high positive correlation between publications and citations (r=0.937, p<0.001).

### Countries/regions and institutions

**Figure 3:**
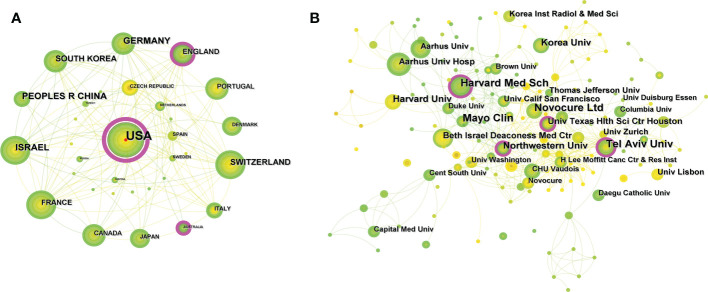
**(A)** Visual mapping of cooperative relationships between countries/regions in related publications. A circle represents a country/region, with larger circles representing more publications and the purple circle on the outer ring representing a higher centrality. **(B)** Visual mapping of collaborative relationships between related publication institutions. A circle represents an institution, the larger the circle means more publication, and the outer purple circle represents having a higher centrality.

Based on the aforementioned search criteria, a total of 248 publications from 32 countries/regions and 221 institutions were obtained after selection. The United States had the largest number of publications (123, 49.60%), followed by Germany (32, 12.90%) and China (30, 12.10%) (**Table 1**). Besides, the country/region collaborations were visualized and analyzed by means of CiteSpace software. The network of collaborative relationships between countries/regions is displayed in **Figure 3A**. Specifically, the United States cooperated most closely with other countries/regions, followed by Germany, Switzerland, China, and South Korea as the most cooperative countries. In addition, the degree of collaboration (DC) between countries/regions was analyzed, which was 25.81%. The research institutions with the largest number of publications were Tel Aviv Univ (10), Harvard Med Sch (10), and Novocure Ltd (10). Among the top 10 institutions, Harvard Med Sch and Univ Texas Hlth Sci Ctr Houston had the highest centrality (both at 0.14), followed by Northwestern Univ (0.11) (**Table 1**). **Figure 3B** shows the visual mapping of research institutions in CiteSpace.

**Table 1:** Top 10 countries/regions and institutions for related publications from 2007 to September 2022.

Rank	Count	Centrality	Year	Countries/regions	Count	Centrality	Year	Institutions
1	123	0.73	2012	USA	10	0.1	2015	Tel Aviv Univ
2	32	0.06	2016	GERMANY	10	0.14	2017	Harvard Med Sch
3	30	0	2019	PEOPLES R CHINA	10	0.07	2015	Novocure Ltd
4	27	0.03	2007	ISRAEL	9	0.04	2017	Mayo Clin
5	21	0.04	2007	SWITZERLAND	7	0.13	2017	Northwestern Univ
6	20	0.01	2016	SOUTH KOREA	7	0	2016	Korea Univ
7	15	0.11	2016	ENGLAND	7	0.03	2013	Harvard Univ
8	14	0.04	2007	FRANCE	6	0.14	2014	Univ Texas Hlth Sci Ctr Houston
9	10	0	2016	CANADA	6	0.02	2014	Beth Israel Deaconess Med Ctr
10	9	0	2014	PORTUGAL	6	0.04	2016	Aarhus Univ Hosp

### Authors and co-cited authors

**Figure 4:**
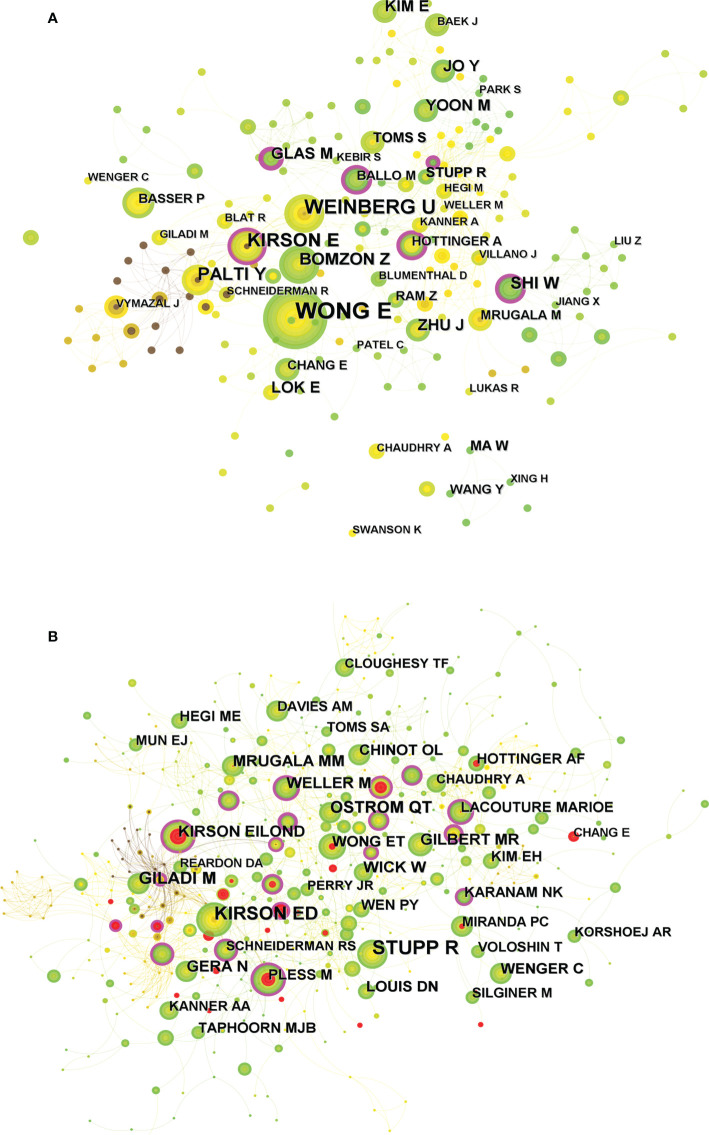
**(A)** Visual mapping of the authors of related publications. A circle represents an author, with larger circles representing more publications by that author, and the purple ring on the outer ring represents having a high centrality. **(B)** Visual mapping of co-cited authors of related publications. A circle represents a co-cited author, with larger circles representing more publications by that co-cited author, and the purple ring on the outer ring represents a high centrality.

A total of 1215 researchers were involved in the publication of relevant literature. WONG E (18) possessed the greatest number of publications, followed by WEINBERG U (11) and KIRSON E (10). Among the top ten authors, SHI W had the highest centrality at 0.12, followed by KIRSON E (0.11) and BOMZON Z (0.1) (**Table 2**). The DC between authors was 97.58%. The visual mapping of the author cooperation network is exhibited in **Figure 4A**. Author Co-citation Analysis (ACA) refers to the condition where two or more authors are cited by one or more papers at the same time, and these authors constitute a co-citation relationship (22). STUPP R (236) was the most cited author, followed by KIRSON ED (164) and GILADI M (104). Among the top ten co-cited authors, KIRSON EILOND and WELLER M had the highest centrality at 0.14 and 0.13, respectively (**Table 2**). The clinical study led by Prof. STUPP on the trial of temozolomide (TMZ) and radiotherapy for glioblastoma showed that the combination of TMZ and radiotherapy resulted in a significant and meaningful survival advantage compared to radiotherapy alone (23). The Stupp treatment protocol of simultaneous radiotherapy and chemotherapy for glioblastoma was later named after Prof. STUPP and is widely used in clinical practice. In addition, Prof. STUPP has led several clinical trials on TTFields therapy for glioblastoma (8, 9), making significant contributions to the advancement of glioblastoma treatment strategies. **Figure 4B** shows a visual network map of the relationship between co-cited authors.

**Table 2:** Top 10 authors and co-cited authors in relevant publications from 2007 to September 2022.

Rank	Count	Centrality	Authors	Rank	Count	Centrality	Co-Cited Authour
1	18	0.07	WONG E	1	236	0.02	STUPP R
2	11	0.05	WEINBERG U	2	164	0.05	KIRSON ED
3	10	0.11	KIRSON E	3	104	0	GILADI M
4	8	0.03	PALTI Y	4	81	0	OSTROM QT
5	7	0.12	SHI W	5	73	0.01	GERA N
6	7	0.1	BOMZON Z	6	71	0.14	KIRSON EILOND
7	6	0.02	JO Y	7	69	0.01	GILBERT MR
8	6	0.02	YOON M	8	63	0.03	WICK W
9	6	0.08	ZHU J	9	63	0.08	MRUGALA MM
10	6	0	LOK E	10	61	0.13	WELLER M

### Journals and co-cited journals

**Figure 5:**
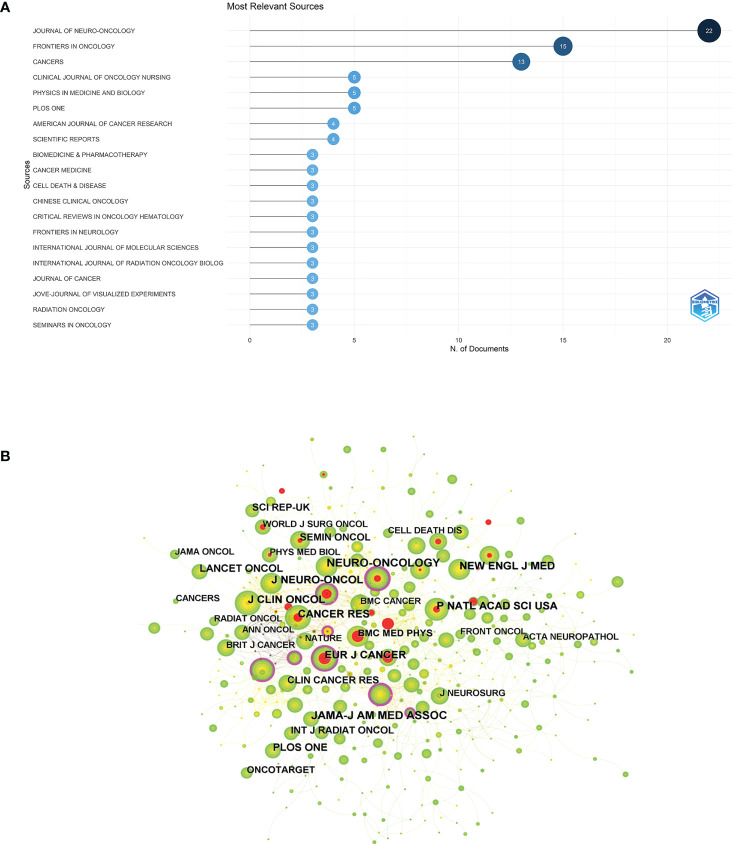
**(A)** Visualization mapping of the top 20 source journals for related publications. **(B)** Visual mapping of co-cited journals of related publications. A circle represents a co-cited journal, the larger the circle the higher the citation frequency of the co-cited journal, and the purple ring of the outer ring represents a high centrality.

The “bibliometrix” package in R software was used to visualize and analyze the journals of the publications. JOURNAL OF NEURO-ONCOLOGY (22) was the most published journal (75), followed by FRONTIERS IN ONCOLOGY (15) and CANCERS (13) (**Table S3**). The visual mapping of the top 20 academic journals in number of publications is illustrated in **Figure 5A**. Among the top 10 journals, CANCERS possessed the highest impact factor (IF) (6.575). JAMA-J AM MED ASSOC was the most cited journal among the 1,832 co-cited journals (205), followed by NEURO-ONCOLOGY (197) (**Table 3**). Moreover, JAMA-J AM MED ASSOC has the highest IF (157.335) and EUR J CANCER had the highest centrality (0.12), indicating that these journals have a great influence in this research field. Journal co-citation reflects the correlation between various journals and disciplines. **Figure 5B** shows the visual mapping of co-cited journals in CiteSpace, where the size of the circle represents the frequency of co-citation, and the purple ring indicates a high centrality. Dual-map overlay of journal is a method to display information about the distribution of articles, citation trajectories, and drift of gravity in each discipline (24). The left side represents the applied citation map and the right side refers to the counterpart cited map. The curve is the citation linkage displaying the citation ins and outs in a complete way. The citation relationships are presented as colored paths in **Figure S2**, where the yellow paths indicate that the publications in molecular/biology/immunology journals are frequently cited by molecular/biology/genetics journals. The green paths indicate that the publications in molecular/medical/clinical journals are frequently cited in molecular/biology/genetics journals.

**Table 3:** Top 10 co-cited journals of related publications.

Rank	Count	Centrality	Cited Journals	JCR	IF 2022
1	205	0.02	JAMA-J AM MED ASSOC	Q1	157.335
2	197	0	NEURO-ONCOLOGY	Q1	13.029
3	176	0	CANCER RES	Q1	13.312
4	170	0.12	EUR J CANCER	Q1	10.002
5	167	0.02	P NATL ACAD SCI USA	Q1	12.779
6	165	0	NEW ENGL J MED	Q1	176.079
7	154	0.02	J NEURO-ONCOL	Q2	4.506
8	150	0.04	J CLIN ONCOL	Q1	50.717
9	132	0.01	PLOS ONE	Q2	3.572
10	124	0.02	LANCET ONCOL	Q1	54.433
11	106	0	SCI REP-UK	Q2	4.996

### Co-cited references and reference burst

**Figure 6:**
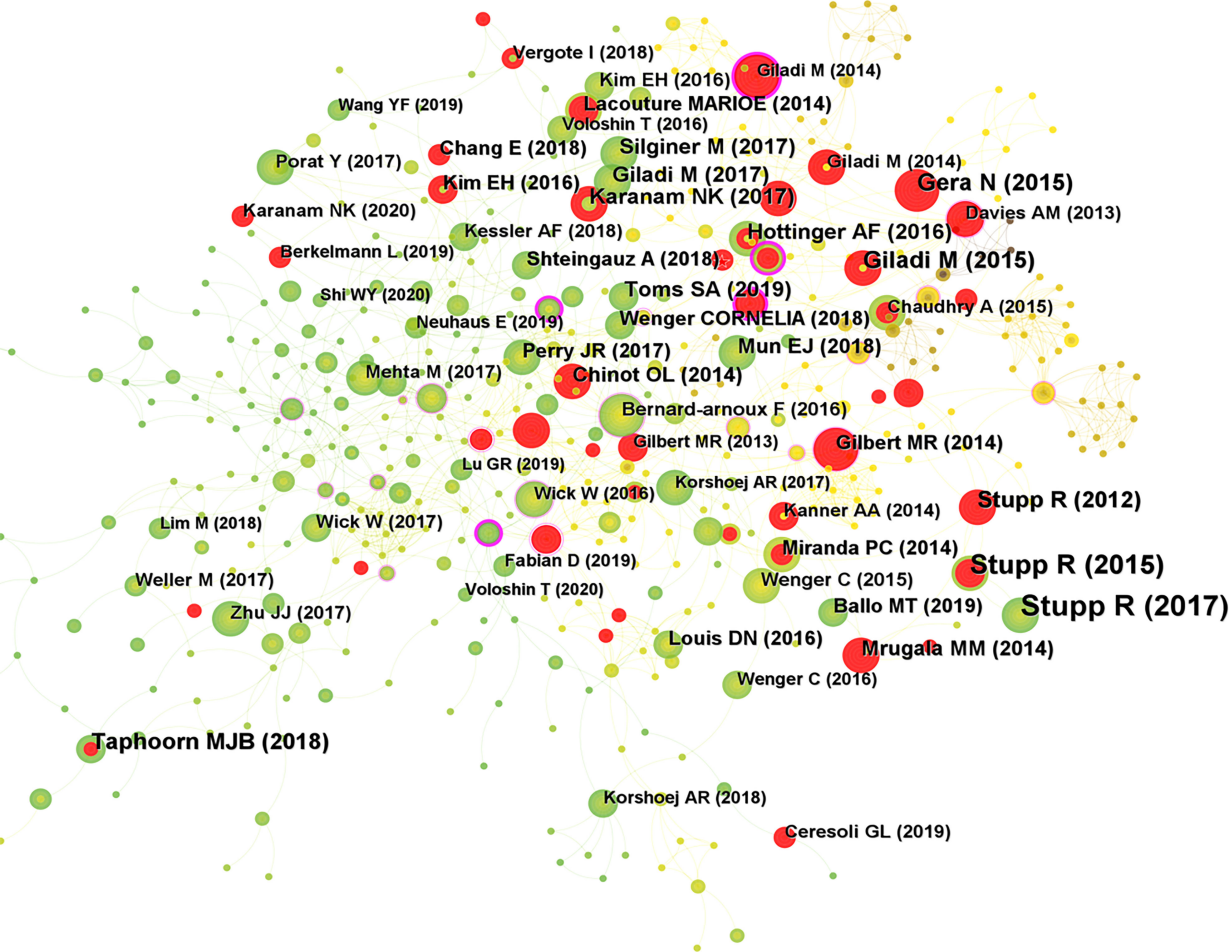
Visual mapping of co-cited references of related publications. A circle represents a co-cited reference, with larger circles representing higher citation frequency of that co-cited reference, with the outer purple ring representing a high centrality and the red circle representing a burst.

Regarding the 500 co-cited references, the top 10 co-cited references are listed in **Table 4**. **Figure 6** shows the visual mapping of the co-cited references. STUPP R et al. (9) published the article “Effect of Tumor-Treating Fields Plus Maintenance Temozolomide *vs.* Maintenance Temozolomide Alone on Survival in Patients With Glioblastoma: A Randomized Clinical Trial” was the most frequently cited (148), which reported the final analysis of a randomized, open-label clinical trial (NCT00916409) on whether TTFields can improve progression-free survival (PFS) and overall survival (OS) in glioblastoma patients. The results showed the combination of TTFields with maintenance TMZ chemotherapy produces a statistically significant improvement in PFS and OS compared with maintenance TMZ alone. According to the titles of the top 10 co-cited references, the research subjects were TTFields Plus maintenance TMZ, TTFields Plus radiotherapy, mitosis, health-related QoL, and cancer treatment. Through citation burst analysis, the topics that have attracted the interest of researchers in a particular research area over time were identifies (25). According to the strongest citation bursts, the first citation burst started in 2009 with a citation burst intensity of 3.92-22.83 for the first 25 references (**Figure S3**).

**Table 4:** Top 10 co-cited references of related publications.

Rank	Count	Centrality	Year	Cited References
1	148	0	2017	Effect of Tumor-Treating Fields Plus Maintenance Temozolomide vs Maintenance Temozolomide Alone on Survival in Patients With Glioblastoma A Randomized Clinical Trial
2	93	0.01	2015	Maintenance Therapy With Tumor-Treating Fields Plus Temozolomide vs Temozolomide Alone for Glioblastoma A Randomized Clinical Trial
3	52	0.05	2015	Tumor Treating Fields Perturb the Localization of Septins and Cause Aberrant Mitotic Exit
4	48	0.01	2015	Mitotic Spindle Disruption by Alternating Electric Fields Leads to Improper Chromosome Segregation and Mitotic Catastrophe in Cancer Cells
5	46	0.05	2012	NovoTTF-100A versus physician’s choice chemotherapy in recurrent glioblastoma: A randomised phase III trial of a novel treatment modality
6	41	0.01	2018	Influence of Treatment With Tumor-Treating Fields on Health-Related Quality of Life of Patients With Newly Diagnosed Glioblastoma A Secondary Analysis of a Randomized Clinical Trial
7	39	0.03	2017	Tumor treating fields (TTFields) delay DNA damage repair following radiation treatment of glioma cells
8	37	0	2016	Tumor treating fields: a novel treatment modality and its use in brain tumors
9	37	0.01	2019	Increased compliance with tumor treating fields therapy is prognostic for improved survival in the treatment of glioblastoma: a subgroup analysis of the EF-14 phase III trial
10	36	0.03	2017	Biological activity of tumor-treating fields in preclinical glioma models
				Tumor-treating fields elicit a conditional vulnerability to ionizing radiation *via* the downregulation of BRCA1 signaling and reduced DNA double-strand break repair capacity in non-small cell lung cancer cell lines
				Tumor-Treating Fields: A Fourth Modality in Cancer Treatment

### Keyword co-occurrence and clustering analyses and burst analysis

In co-occurrence analysis, the co-occurrence of lexical pairs or noun phrases in a collection of literature is utilized to determine the relationships between topics in the disciplines represented by that collection (26). Generally, it is considered that if the same term occurs more frequently in two documents, the relationship between the two subjects will be closer (27). By counting the frequency of keywords appearing in the same document, a co-word network can be formed, and the proximity of the nodes in the network can reflect the closeness of the subject content. The top 10 keywords in the literature related to the TTFields therapy for glioblastoma are shown in **Table S4**, including glioblastoma (156), tumor treating field (152), temozolomide (134), randomized phase III (48), brain (46), survivor (46), cancer (44), trial (42), alternating electric field (42), and radiotherapy (36), indicating that these fields are currently the hot directions of research on TTFields therapy for glioblastoma.

**Figure 7:**
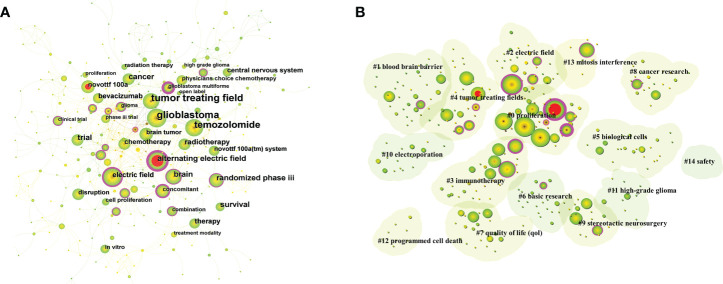
**(A)** Visual mapping of keyword co-occurrence for related publications. A circle represents a keyword, the larger the circle means the higher the frequency of that keyword, the purple ring of the outer ring represents having a high centrality. **(B)** Visual mapping of keyword clusters for related publications. Each cluster is a composition of multiple closely related words, forming a total of 15 clusters, with smaller numbers representing more keywords contained in that cluster.

There were 321 nodes with 761 links in the keyword co-occurrence network map (**Figure 7A**), each of which corresponded to a keyword, and the larger node size indicated a higher frequency. The number of links between nodes and the distance between nodes reflect the closeness of the keywords. The purple circles outside the nodes stood for higher centrality, and the red circles inside the nodes meant higher prominence intensity. Further clustering analysis of keywords based on the CiteSpace keyword co-occurrence analysis can reflect the hot directions of this research field. **Figure 7B** shows the visual mapping of 15 keyword clusters, mainly including proliferation, blood brain barrier, electric field, immunotherapy, tumor treating fields, biological cells, basic research, quality of life (QoL), cancer research, stereotactic neurosurgery, electroporation, high-grade glioma, programmed cell death, mitosis interference, and safety.

Furthermore, keyword burst analysis was conducted to find out the phase of research hotspots and the duration of the related fields. The burst intensity of the top 15 keywords is displayed in the keyword burst plot (**Figure S4**), in which the first burst started in 2007, and the burst intensity ranged from 1.44 to 5.88. Additionally, alternating electric field (5.88) had the highest burst intensity. The length of the red line represented the duration of the burst, while phase II trial and alternating electric field were the keywords with the longest duration of burst.

## Discussion

### General information

To some extent, the publication time and annual volume distribution of the literature can reflect the research hotspot and development rate of the research field more intuitively. As shown in **Figure 2**, the annual and cumulative numbers of publications related to TTFields therapy for glioblastoma were both on the rise, with an AGR of 17.41% during 2007 and 2021, indicating that the research on TTFields therapy for glioblastoma is receiving more and more attention and importance from scholars, and their research enthusiasm is increasing. The number and centrality of publications by countries/regions and institutions can objectively reflect the level and influence of scientific research in relevant research field. The United States had the largest number of publications (123, 49.60%), followed by Germany (32, 12.90%) and China (30, 12.10%), indicating that these countries are the main scientific exporters in this research field and have made great contributions to the development of TTFields therapy for glioblastoma. The analysis of the network of international collaborative relationships by countries/regions manifested that the United States and Germany collaborated most closely with other countries. The DC of countries/regions was 25.81%. Therefore, it is necessary to strengthen the research cooperation relationship between countries/regions in future research and enhance international exchange to promote the development of the field.

WONG E (18) published the largest number of publications, followed by WEINBERG U (11) and KIRSON E (10). Among the top ten authors, SHI W had the highest centrality (0.12), followed by KIRSON E (0.11) and BOMZON Z (0.1), suggesting their great contribution to the development of the field. The inter-author DC was 97.58%. Wong Et al. (28) reported a profound effect of dexamethasone on both TTFields therapy and chemotherapy, leading to a reduced OS in glioblastoma patients. Thus, the full immunosuppression of dexamethasone may interfere with the immune function required to treat glioblastoma. According to previous trials, array-related skin irritation was the most common adverse event (AE) associated with TTFields therapy. Shi W et al. (29) reported on real-world AEs associated with TTFields therapy in a clinical practice setting. No new safety issues were identified in a safety monitoring analysis for TTFields therapy involving more than 11,000 patients, with a favorable safety profile comparable to the published TTFields/glioblastoma trial, implying its feasibility in multiple populations, including the elderly patients.

STUPP R was the most cited author (236), followed by KIRSON ED (164) and GILADI M (104). Among the top ten co-cited authors, KIRSON EILOND and WELLER M had the highest centrality at 0.14 and 0.13, respectively. The clinical trial led by Prof. STUPP reported that the combination of TMZ and radiotherapy produced a significant and meaningful survival benefit for glioblastoma patients, and such a combination regimen is named as Stupp protocol (21). The Stupp treatment protocol is still widely used in clinical practice, serving as the standard of care for glioblastoma patients after surgery. In addition, a randomized clinical trial reported the efficacy of maintenance treatment with TTFields therapy plus TMZ *vs.* TMZ alone in glioblastoma (8), and interim analysis demonstrated that adding TTFields to maintenance TMZ chemotherapy significantly prolonged the PFS and OS. Besides, the final analysis of this randomized clinical trial also illustrated that the combination of TTFields therapy with maintenance TMZ chemotherapy resulted in statistically significant improvements in the PFS and OS compared with maintenance TMZ alone (9). These results are consistent with a previous interim analysis. Kirson ED et al. (30) reported that low-intensity, intermediate-frequency, electric fields inhibit cancer cell growth *in vitro* through an anti-microtubule mechanism of action. TTFields is a safe and effective new treatment modality that efficiently slows tumor growth *in vitro* and *in vivo*, and in human cancer patients as well. Giladi M et al. (13) reported that the effect of TTFields on cell viability and colony survival depends mainly on the cell division rate. The slowly dividing cells can be affected by extending the exposure time to TTFields to a similar extent as the rapidly dividing cells. Giladi M et al. (31) evaluated the efficacy of TTFields combined with radiotherapy on glioma cells and found that TTFields synergistically enhanced the efficacy of radiotherapy on glioma cells. The use of TTFields after radiotherapy may improve the effect on glioma cells by inhibiting DNA damage repair, and TTFields therapy may be a viable strategy to improve the efficacy of radiotherapy.

JOURNAL OF NEURO-ONCOLOGY (22) was the most published journal, followed by FRONTIERS IN ONCOLOGY (15) and CANCERS (13). CANCERS displayed the highest IF (6.575) among the top 10 academic journals, denoting the influence of these journals in the glioblastoma treatment using TTFields. Based on the dual-map overlay of journals, the yellow paths stood for the publications in molecular/biology/immunology journals that are frequently cited by molecular/biology/genetics journals. The green paths indicated that the publications in molecular/medical/clinical journals are frequently cited in molecular/biology/genetics journals.

As the most critical part of the co-citation analysis, the co-cited reference analysis explores the information of the top 10 co-cited literature that can be considered as the classic literature in the field. The co-citation analysis of references can reveal the research subjects clustered in the field. According to the top 10 co-cited references, the research directions in this field are mainly about TTFields therapy combined with TMZ chemotherapy, TTFields therapy combined with radiotherapy, mitosis, health-related QoL, and cancer treatment.

The strongest citation burst was caused by STUPP R et al. (32) who reported the first controlled trial of a novel cancer treatment modality combined with electric fields rather than chemotherapy. NovoTTFIELDS-100A is a portable device that delivers low-intensity, intermediate-frequency electric fields through a non-invasive array of transducers. The results of a phase III trial of chemotherapy-free treatment with NovoTTFIELDS *vs.* active chemotherapy for patients with recurrent glioblastoma showed that despite unimproved OS, the efficacy and activity of this chemotherapy-free treatment device appeared to be comparable to those of chemotherapy regimens typically used for recurrent glioblastoma. Toxicity and QoL clearly favored TTFields. Based on the results of this research trial, TTFields therapy was approved for the treatment of recurrent glioblastoma. In addition, the general anti-cancer effect of TTFields may be applicable to other types of solid tumors, alone or in combination with chemotherapy. In particular, such a non-invasive treatment may provide clinical benefit and greatly expand the therapeutic armamentarium for tumors with severe local lesion burdens and poor systemic conditions where further radiotherapy or chemotherapy is not an option. In 3 references that have continued from the 2020 burst to the present, Chang E et al. (33) reported that TTFields altered the cell membrane structure, resulting in greater permeability to chemotherapeutic agents. The other 2 references are clinical trial studies on TTFields combined with chemotherapy for the treatment of malignancies (34, 35).

### Research status and hotspots

As the core summary of research content, keyword analysis is an important method to uncover research hotspots and development lines. The top 10 keywords in publications related to TTFields therapy for glioblastoma included glioblastoma (156), tumor treating field (152), temozolomide (134), randomized phase III (48), brain (46), survival (46), cancer (44), trial (42), alternating electric field (42), and radiotherapy (36). Keyword co-occurrence analysis was performed to reveal the relationship between research topics according to the frequency and circumstances of keyword co-occurrence. A cluster analysis was performed based on the keyword co-occurrence analysis, which resulted in 15 clusters involving proliferation, blood brain barrier, electric field, immunotherapy, tumor treating fields, biological cells, basic research, quality of life (QoL), cancer research, stereotactic neurosurgery, electroporation, high-grade glioma, programmed cell death, mitosis interference, and safety, thereby identifying the current research hotspots and possible future trends in the field of glioblastoma. The main contents are as follows.

(1) Mechanism of TTFields therapy

TTFields represents an emerging non-invasive anti-cancer treatment approach involving transdermal delivery of low-intensity (1-3 V/cm), intermediate-frequency (100-300 kHz), alternating electric fields (it is also referred to as alternating electric field therapy), which exerts biophysical forces on charged and polarized molecules called dipoles (36). Kirson ED et al. (11) reported that low-intensity, intermediate-frequency (100-300 kHz), alternating electric fields delivered by insulated electrodes have profoundly inhibited the growth rates of various human and rodent tumor cell lines as well as animal malignancies. This discovery demonstrates the potential applicability of electric fields as a novel treatment modality for malignant tumors. TTFields was initially discovered to inhibit cancer cell proliferation by interfering with the mitotic apparatus. However, a growing number of studies have demonstrated that TTFields possesses a broad range of anti-tumor mechanisms of action, such as disrupting multiple biological processes (e.g. DNA repair, cell permeability and immune response) to produce therapeutic effects.

Several hypotheses have been proposed to explain the basis of anti-cancer action mechanism of TTFields. A common denominator of these hypotheses is the assumption that electric fields exert directional forces on intracellular polar elements, such as organelles and macromolecules. The mitotic cells with shuttle-shaped microtubules may become susceptible to externally applied electric fields because of the presence of highly polar and dynamic structural features (37, 38). TTFields interferes with the normal formation of the mitotic spindle, eventually activating the spindle assembly checkpoint (SAC) and consequently triggering apoptosis in a manner similar to that observed in studies with classical anti-microtubule agents (39, 40). In addition, glioblastoma cell proliferation is effectively reduced through a key SAC regulator, namely, monopolar spindle 1 (MPS-1, also known as TTK) (41), an evolutionarily conserved protein kinase inhibiting the SAC from binding to spindle toxin (42). The mitotic spindle controls the necessary capture, alignment and segregation of chromosomes in the two daughter cells (43). The SAC initiates mitotic cell cycle arrest by blocking the progression from mitosis to anaphase (44), and defects in the SAC lead to chromosomal instability, aneuploidy and subsequent tumorigenesis (45). It has been reported that TTFields can reduce glioblastoma cell proliferation and increase apoptosis in combination with chemosuppression of SAC, with the potential as a bridge to clinical disruption of TTFields therapy (46).

It has been revealed that TTFields is able to not only reduce the re-association of radiation-induced DNA double-strand breaks (DSBs) but also induce DNA DSBs. The mechanism by which TTFields produces DNA DSBs is correlated with the production of replication stress, including the DNA replication complex genes MCM6 and MCM10 (47). TTFields has also been proven to trigger the generation of autophagosome dependent on adenosine monophosphate-activated protein kinase by interfering with DNA fork replication (47, 48), targeting DNA damage repair and the breast cancer 1 (BRCA1)-mediated homologous recombination pathway, and inducing endoplasmic reticulum stress during mitosis through increasing the lipidation of protein light chain 3 α/β-I (LC3A/B-I) to form LC3A/B-II (49). Kim EH et al. (50) demonstrated that TTFields therapy up-regulates autophagy-related genes and induces cell morphological changes, and that TTFields-induced autophagy in glioblastoma is associated with reduced Akt2 expression *via* the mTOR/p70S6K pathway. Autophagy is identified as a key cell death pathway triggered by TTFields in glioblastoma, suggesting that TTFields is a potential therapeutic option for glioblastoma. Therefore, understanding the exact mechanism of TTFields in triggering cell death can provide better insights for improved therapies and combination therapies.

TTFields also alters the cell membrane structure to be more permeable to chemotherapeutic agents. Chang E et al. (33) argued that TTFields exposure induces increased number and size of pores in glioblastoma cell membrane. When TTFields was applied, the morphology of glioblastoma cell membranes was also disturbed. Furthermore, the effect of TTFields on glioblastoma cell membrane permeability was reversible after TTFields exposure was stopped. Interestingly, TTFields exposure results in a significant increase in 5-aminolevulinic acid (5-ALA) uptake in glioblastoma cells compared to that in fibroblasts (51). 5-ALA is approved by the FDA for clinical use in the United States to assist neurosurgeons in delineating the boundary between tumor and normal brain tissue during glioma resection (52). Pretreatment of glioma patients with TTFields prior to 5-ALA administration can be considered in future clinical studies, which may enhance the display of invasive tumor margins during tumor resection.

Several studies have explored the correlation between TTFields and immune pathways, and TTFields-induced cell death can stimulate immune effects. For example, Voloshin T et al. (53) reported that TTFields therapy promotes phagocytosis of cancer cells through the maturation of dendritic cells (DCs) and the recruitment of immune cells *in vivo*. It has been indicated in animal models of solid tumors that TTFields can stimulate immunogenic cell death and promote immune cell recruitment (12, 53), thus increasing the hope that TTFields will reverse local and systemic immunosuppressive stimuli in glioblastoma patients. Recently, Chen et al. (54) explored the role of TTFields in reinvigorating immune response, and demonstrated that TTFields promotes the production of immunostimulatory and pro-inflammatory interferon type 1 cytokines in tumor cells through a cGAS/STING and AIM2 inflammatory vesicle-dependent mechanism, ultimately activating the immune system. The results showed that TTFields not only directly inhibits tumor cell growth but also enhances anti-tumor immunity, as previously described, suggesting that TTFields can be used as an immunomodulatory approach for glioblastoma. However, this study focused only on peripheral immune responses, and future studies need to address whether TTFields can reinvigorate most of the suppressed anti-tumor immunity in the tumor microenvironment (TME) (12). Bezu L et al. (55) hypothesized that TTFields-induced endoplasmic reticulum stress is only at the level of phosphorylation of eIF2α, a marker of immunogenic cell death. In the future, more clinical studies are needed to evaluate the correlation between TTFields and immunity. Overall, as a potential therapeutic strategy for immunotherapy, TTFields is available for glioblastoma and other solid tumors.

(2) Research application of TTFields to glioblastoma

TTFields therapy is actually performed continuously (>18 h/day) *via* an array of four transducers placed on the shaved scalp with minimal surface resistance of glioblastoma patients, and then the transducers are connected to a portable TTFields medical device that delivers low-intensity, intermediate-frequency, electric fields *via* the non-invasive array of transducers (32, 56). Jo Y et al. (57) evaluated the biological effectiveness of fractionated treatment protocol using TTFields in cancer. The results showed that the effect on tumor cells is more prominent than that on normal cells during the treatment time of 3 to 12 h/d, while the difference is minimal during the treatment time of 24 h/d. Such a protocol shortens the treatment time while maintaining the efficacy, suggesting that this method may be applicable to cancer treatment. In an open prospective pilot study, Salzberg M et al. (58) evaluated the safety, tolerability and efficacy of the NovoTTFIELDS100A(TM) device for the treatment of patients with locally advanced and/or metastatic solid tumors. All six patients in the study showed good tolerability without associated serious AEs, demonstrating the potential of TTFields as a new treatment modality for solid tumors that definitely warrants further investigation. TTFields was initially shown to be effective in blocking the proliferation and inducing the death of various tumor cells in culture as well as solid tumors in animals. On this basis, Kirson ED et al. (30) initiated a pilot clinical trial to explore the effectiveness of TTFields in 10 patients with recurrent glioblastoma and demonstrated that TTFields inhibits the growth of this highly treatment-resistant tumor by virtue of special insulated electrodes with few side effects. The results of this study also evidence the safety and efficacy of TTFields for the treatment of cancer patients for the first time.

Based on the results of the EF-11 trial, TTFields was first approved in 2011 for histologically or radiologically confirmed glioblastoma that had relapsed after standard-of-care chemotherapy (59). An international randomized phase III clinical trial, EF-14, was subsequently conducted to include patients with newly diagnosed glioblastoma into one of the two groups: TTFields plus TMZ *vs.* TMZ alone (8). Compared to those in the TMZ alone group, the patients in the TTFields plus TMZ group showed improved PFS (7.1 *vs.* 4.0 months) and two-year survival rates (43% *vs.* 95%), and no significant additional toxicity or AE was found in the TTFields plus TMZ group. Afterwards, TTFields in combination with TMZ was approved by the FDA for the treatment of newly diagnosed and histologically confirmed glioblastomas after maximal reduction surgery and completed radiotherapy and standard-of-care chemotherapy (60). Voloshin T et al. (53) reported that TTFields therapy can induce anti-tumor immune responses and demonstrated the powerful efficacy of simultaneous application of TTFields and anti-(programmed death-1) PD-1 therapies. Combining TTFields with anti-PD-1 therapies can further enhance anti-tumor immunity, resulting in better tumor control. Glas M et al. (61) reported a direct correlation between TTFields dose distribution and tumor response, confirming the therapeutic activity of TTFields and the reason for optimizing array placement to maximize the TTFields dose in regions with the highest risk of progression.

Currently, preclinical studies on TTFields are ongoing in several types of cancers, including breast cancer, cervical cancer, colorectal cancer, gastric cancer, hepatocellular carcinoma, melanoma, small cell lung cancer, and other solid tumors (56). These preclinical studies and subsequent clinical trials will help determine the feasibility of applying TTFields to cancer treatment in a more general way in the future. As new cancers in different anatomical regions are considered, it is also an important challenge to design new model types and dressers with optimal functionality and user-friendliness. To date, however, TTFields has not been studied in hematologic tumors yet. In addition, unlike systemic chemotherapy, the delivery of TTFields can be targeted locally, thereby minimizing the risk of systemic adverse effects, which may allow TTFields to be combined with other anti-cancer therapies for enhanced efficacy without increasing the toxicity. Several preclinical studies suggest that TTFields may act in an additive or synergistic manner with certain cytotoxic drugs, and when used in combination with immune checkpoint inhibitors, it can promote the death of immunogenic cells. The positive effects of TTFields, as a truly novel cancer treatment modality, alone or in combination with other therapeutic approaches, already observed in glioblastoma will be multiple useful indications.

Limitations: 1) The relevant literature retrieved was only from the WoSCC database and in English language, so literature from other database sources and in other types of languages may be missed. 2) The literature from the WoSCC database is continuously updated, while the data we collected was only from 2007 to September 2022. 3) Manual removal of irrelevant literature to the study by the reviewer may lead to selection bias.

## Conclusions

In this study, bibliometric tools were utilized to analyze publications in the field of TTFields therapy for glioblastoma, revealing relevant bibliometric characteristics of the field. A comprehensive analysis of publications in this field from 2007 to September 2022 was performed to identify the current status of research and research trends. TTFields, as the fourth therapeutic approach for the treatment of glioblastoma characterized by local delivery and low toxicity, highlights the potential for tumor control and therapeutic effect on target organs.

## Author contributions

XD, CC conceived the study and performed the literature search, data processing, and preparation of figures and tables. XD drafted the manuscript. YX, YC, LY, XL, XL, RW revised the manuscript. BT supervised and revised the manuscript. All authors contributed to the article and approved the submission of the manuscript.

## Acknowledgments

The first author would like to thank her beloved for her company and support.

## Conflict of interest

The authors declare that the research was conducted in the absence of any commercial or financial relationships that could be construed as a potential conflict of interest.

## Publisher’s note

All claims expressed in this article are solely those of the authors and do not necessarily represent those of their affiliated organizations, or those of the publisher, the editors and the reviewers. Any product that may be evaluated in this article, or claim that may be made by its manufacturer, is not guaranteed or endorsed by the publisher.
