# Supplementary material for: Research on application of tumor treating fields in glioblastoma: A bibliometric and visual analysis
Source: Front Oncol. 2022 Nov 10;12:1055366. doi: 10.3389/fonc.2022.1055366 (PMC9684468; doi:10.3389/fonc.2022.1055366)
Supplement: Supplementary file 1 [file DataSheet_1.pdf]

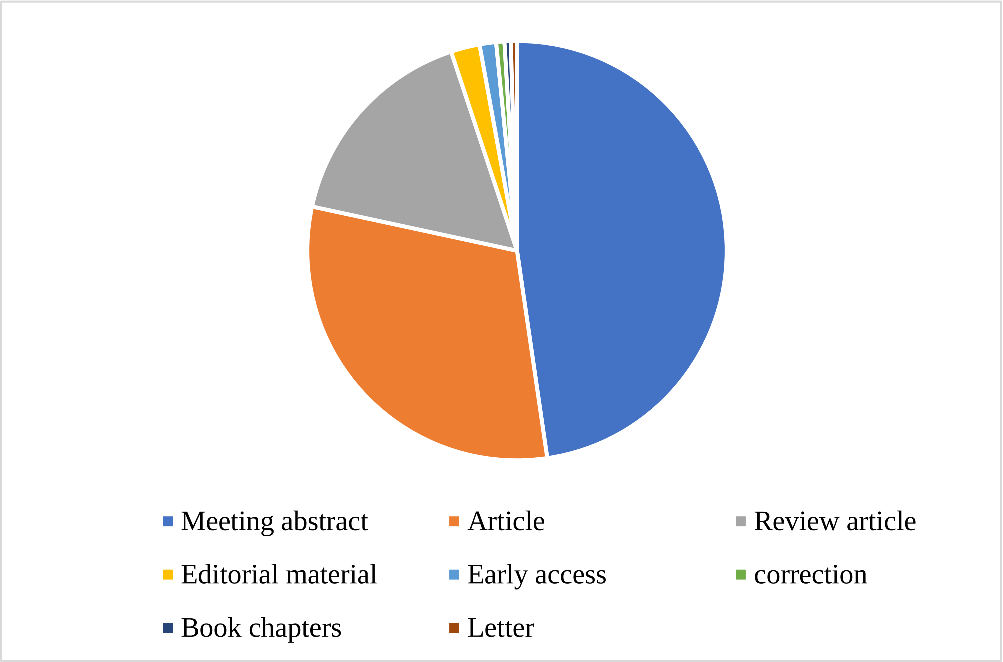

Figure S1A. Related publications pie chart of literature types.

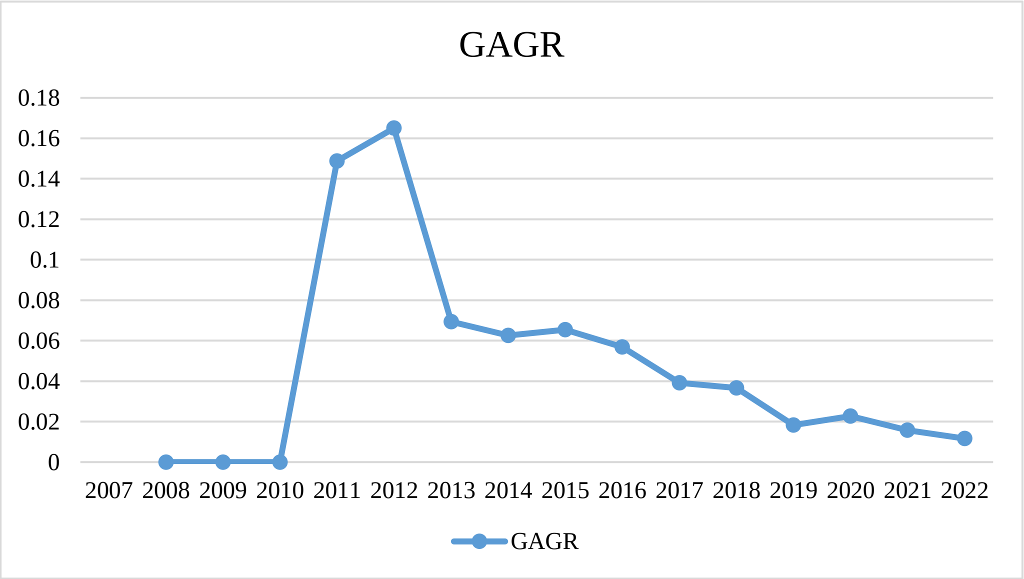

Figure S1B. Compound annual growth rate (GAGR) of publications form 2007 to September 2022.

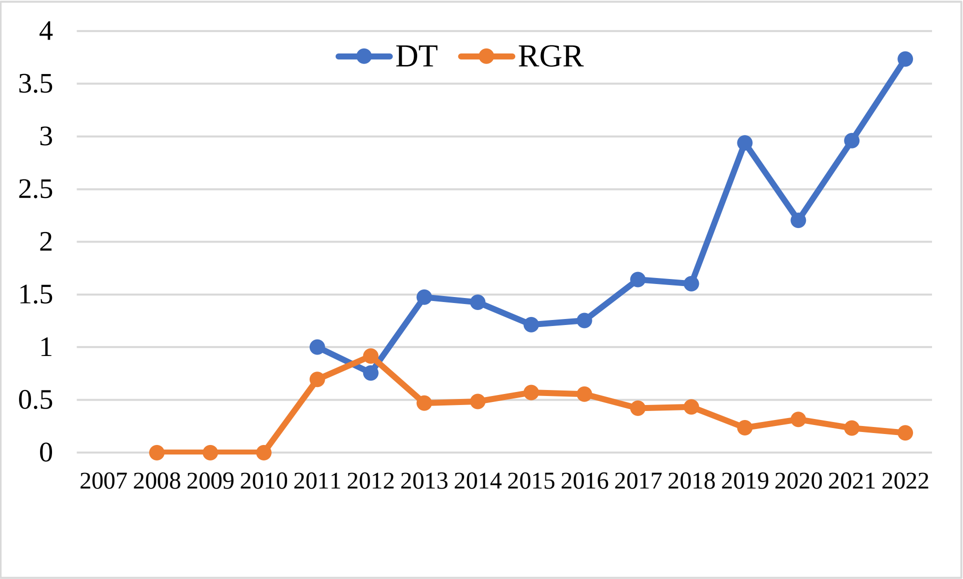

Figure S1C. Relative growth rate (RGR) and doubling time (DT) of publications form 2007 to September 2022.

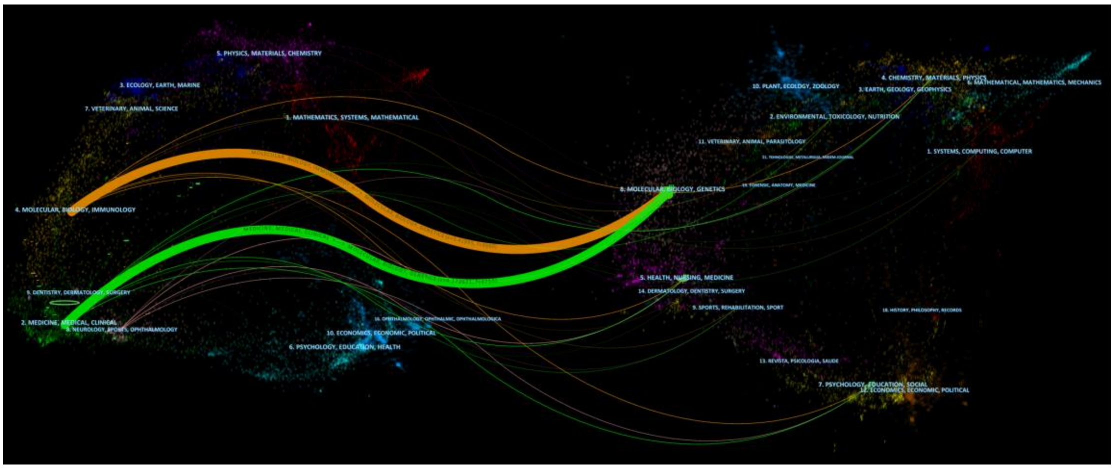

Figure S2. The dual-map overlay of journals of related publications.

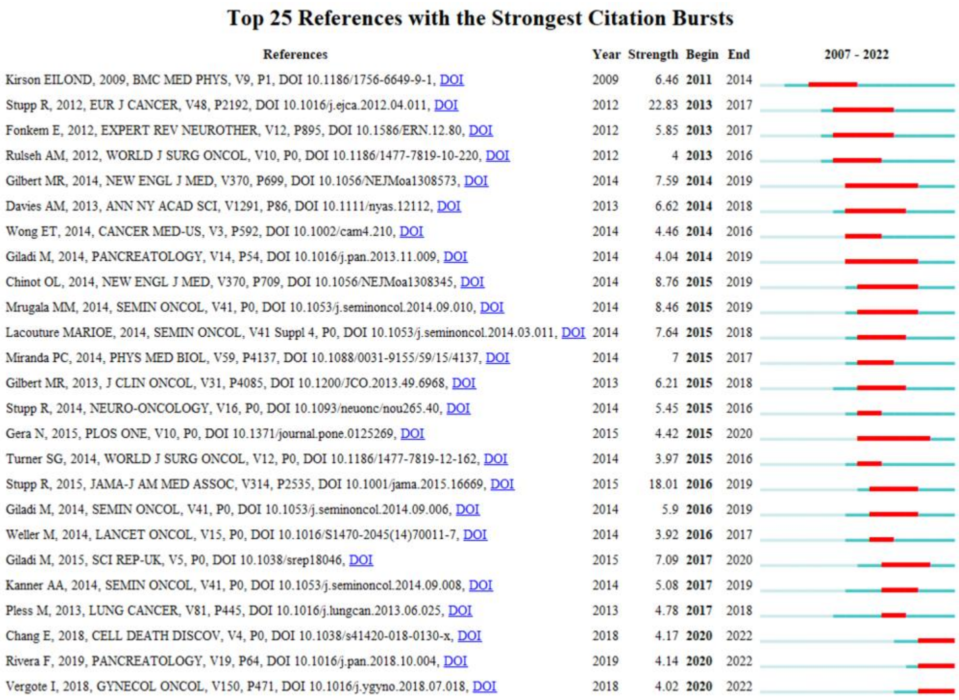

Figure S3. Visualization map of top 25 references with the strongest citation bursts.

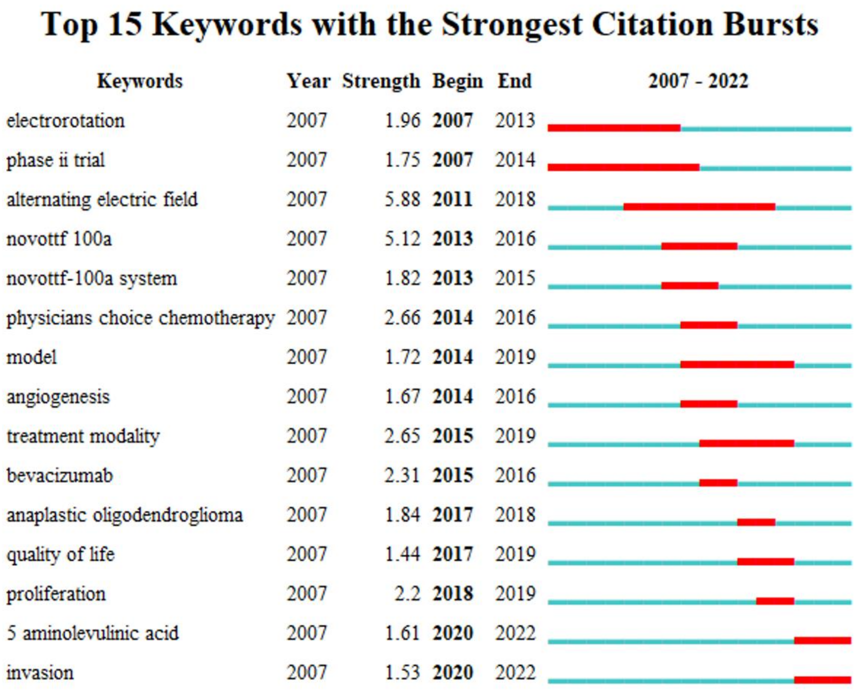

Figure S4. Top 15 keywords with the strongest citation bursts for related publications.
