# Supplementary material for: Research on application of tumor treating fields in glioblastoma: A bibliometric and visual analysis
Source: Front Oncol. 2022 Nov 10;12:1055366. doi: 10.3389/fonc.2022.1055366 (PMC9684468; doi:10.3389/fonc.2022.1055366)
Supplement: Supplementary file 2 [file DataSheet_2.pdf]

Table S1. Compound annual growth rate（GAGR） of publications from 2007 to September 2022

year	Number of publications	Cumulative total	GAGR
2007	1	1	
2008	0	1	0
2009	0	1	0
2010	0	1	0
2011	1	2	14.87%
2012	3	5	16.50%
2013	3	8	6.94%
2014	5	13	6.26%
2015	10	23	6.54%
2016	17	40	5.69%
2017	21	61	3.91%
2018	33	94	3.67%
2019	25	119	1.83%
2020	44	163	2.27%
2021	43	206	1.57%
2022	42	248	1.17%

Table S2. Relative growth rate (RGR) and doubling time (DT) of publications from 2007 to September 2022

year	Number of publications	Cumulative total	RGR	DT
2007	1	1		
2008	0	1	0	
2009	0	1	0	
2010	0	1	0	
2011	1	2	69.31%	1.00
2012	3	5	91.63%	0.76
2013	3	8	47.00%	1.47
2014	5	13	48.55%	1.43
2015	10	23	57.05%	1.21
2016	17	40	55.34%	1.25
2017	21	61	42.20%	1.64
2018	33	94	43.24%	1.60
2019	25	119	23.58%	2.94
2020	44	163	31.46%	2.20
2021	43	206	23.41%	2.96
2022	42	248	18.56%	3.73

Table S3. Top 10 source journals for related publications

Rank	Count	Sources Journal	IF 2022	JCR
1	22	JOURNAL OF NEURO-ONCOLOGY	4.506	Q2
2	15	FRONTIERS IN ONCOLOGY	5.738	Q2
3	13	CANCERS	6.575	Q1
4	5	CLINICAL JOURNAL OF ONCOLOGY NURSING	1.283	Q4
5	5	PHYSICS IN MEDICINE AND BIOLOGY	4.174	Q2
6	5	PLOS ONE	3.572	Q2
7	4	AMERICAN JOURNAL OF CANCER RESEARCH	5.942	Q2
8	4	SCIENTIFIC REPORTS	4.996	Q2
9	3	BIOMEDICINE & PHARMACOTHERAPY	7.419	Q1
10	3	CANCER MEDICINE	4.711	Q2

Table S4. Top 10 keywords for related publications

Rank	Count	Centrality	Keywords
1	156	0.01	glioblastoma
2	152	0	tumor treating field
3	134	0.03	temozolomide
4	48	0.12	randomized phase iii
5	46	0.13	brain
6	46	0.03	survival
7	44	0.03	cancer
8	42	0.03	trial
9	42	0.49	alternating electric field
10	36	0.07	radiotherapy
